# Supplementary material for: Finite temperature quantum annealing solving exponentially small gap problem with non-monotonic success probability
Source: Nat Commun. 2018 Jul 25;9:2917. doi: 10.1038/s41467-018-05239-9 (PMC6060131; doi:10.1038/s41467-018-05239-9)
Supplement: Supplementary file 1 — Supplementary Information [file 41467_2018_5239_MOESM1_ESM.pdf]

Supplementary Information —

Finite temperature quantum annealing solving exponentially small gap problem with non-monotonic success probability

Mishra et. al.

Supplementary Note 1. JORDAN-WIGNER TRANSFORM

The Jordan-Wigner transform can be used to map a one-dimensional transverse field Ising Hamiltonian to a Hamiltonian of uncoupled (free) fermions. A system of dimension $2^N \times 2^N$ is thus effectively reduced to a system of dimension N, which is essential for our simulations involving N as large as 200. We briefly summarize the approach found in the classic work of Lieb *et al.* [1].

We start by defining fermionic raising operators $a_i^\dagger = \frac{1}{2}\left(\otimes_{j=1}^{i-1}\sigma_j^x\right)\left(\sigma_i^z + i\sigma_i^y\right)$ $(i = 1, \ldots, N)$ and their corresponding lowering operators a_i. It is easy to check that the fermionic canonical commutation relations are then satisfied: $\{a_i, a_j\} = \{a_i^\dagger, a_j^\dagger\} = 0$ and $\{a_i^\dagger, a_j\} = \delta_{ij}$. Rewriting Article's Eq. (1) as

$$H = -\Gamma \sum_{i=1}^{N} \sigma_i^x - \sum_{i=1}^{N-1} J_i \sigma_i^z \sigma_{i+1}^z \tag{1}$$

and substituting $\sigma_i^x = 1 - 2a_i^\dagger a_i$, $\sigma_i^z\sigma_{i+1}^z = (a_i^\dagger - a_i)(a_{i+1}^\dagger + a_{i+1})$ gives

$$H = -N\Gamma + \sum_{ij} a_i^\dagger A_{ij} a_j + \frac{1}{2}(a_i^\dagger B_{ij} a_j^\dagger + a_i B_{ji} a_j) \ , \tag{2}$$

or

$$H = -N\Gamma + (\mathbf{a}^\dagger)^T A \mathbf{a} + \frac{1}{2}\left[(a^\dagger)^T B a^\dagger + \mathbf{a}^T B^T \mathbf{a}\right] \tag{3}$$

where $(\mathbf{a}^\dagger)^T = (a_1^\dagger, \ldots, a_N^\dagger)$, $\mathbf{a}^T = (a_1, \ldots, a_N)$, and

$$A = \begin{pmatrix} 2\Gamma & -J_1 & 0 & & \\ -J_1 & 2\Gamma & -J_2 & & \\ 0 & -J_2 & \ddots & \ddots & 0 \\ & & \ddots & 2\Gamma & -J_{N-1} \\ & \cdots & & -J_{N-1} & 2\Gamma \end{pmatrix} \ , \tag{4}$$

$$B = \begin{pmatrix} 0 & -J_1 & & & \\ J_1 & 0 & -J_2 & & \\ & J_2 & \ddots & \ddots & 0 \\ & & \ddots & 0 & -J_{N-1} \\ & \cdots & & J_{N-1} & 0 \end{pmatrix} \ . \tag{5}$$

The Hamiltonian in Supplementary Eq. (2) is not fermion number conserving (it contains terms such as $a_i^\dagger a_j^\dagger$, which means that $\sum_i \sigma_i^x$ is not conserved), but it can be diagonalized by a Bogoliubov transformation [1, Appendix A] in terms of a new set of fermionic operators $\{\eta_i, \eta_i^\dagger\}$:

$$H = E_\mathrm{g} + \sum_{i=1}^{N} \lambda_i \eta_i^\dagger \eta_i \ . \tag{6}$$

where the λ_i are the single-fermion energies of the system. The new fermionic operators are real linear combinations of the old ones:

$$\begin{aligned} \eta_i^\dagger &= \sum_k g_{ik} a_k^\dagger + \sum_k h_{ik} a_k \\ \eta_i &= \sum_k g_{ik} a_k + \sum_k h_{ik} a_k^\dagger \ . \end{aligned} \tag{7}$$

From Supplementary Eq. (6) we get

$$[\eta_k, H] = \lambda_k \eta_k \ . \tag{8}$$

Substituting Supplementary Eqs. (2) and (7) in Supplementary Eq. (8) and setting the coefficients of every operator $\{a_i, a_i^\dagger\}$ to zero gives

$$\begin{aligned} \lambda_k g_{ki} &= \sum_j g_{kj} A_{ji} - h_{kj} B_{ji} \\ \lambda_k h_{ki} &= \sum_j g_{kj} B_{ji} - h_{kj} A_{ji} \end{aligned} \tag{9}$$

Let $\phi_{ik} = g_{ik} + h_{ik}$ and $\psi_{ik} = g_{ik} - h_{ik}$. Plugging this into Supplementary Eq. (9), we get two coupled equations:

$$\mathbf{\Phi}_k(A - B) = \lambda_k \mathbf{\Psi}_k \tag{10}$$
$$\mathbf{\Psi}_k(A + B) = \lambda_k \mathbf{\Phi}_k \ , \tag{11}$$

where $\mathbf{\Phi}_k = (\phi_{1k}, \ldots, \phi_{Nk})$ and $\mathbf{\Psi} = (\psi_{1k}, \ldots, \psi_{Nk})$. Eliminating $\mathbf{\Psi}_k$ gives us the decoupled equation,

$$\mathbf{\Phi}_k(A - B)(A + B) = \lambda_k^2 \mathbf{\Phi}_k \ . \tag{12}$$

Solving the eigensystem given by Supplementary Eq. (12) gives the eigenvalues $\{\lambda_i\}$ in the Hamiltonian (6).

We can find the ground state energy E_g by taking trace of Supplementary Eqs. (2) and (6) [1, Appendix A]. From Supplementary Eq. (2), we have

$$\mathrm{Tr}(H) = 2^{N-1} \sum_i A_{ii} - 2^N N\Gamma \tag{13}$$

and from Supplementary Eq. (6) we have,

$$\mathrm{Tr}(H) = 2^{N-1} \sum_k \lambda_k + 2^N E_\mathrm{g} \ . \tag{14}$$

As the trace is invariant under a canonical transform, we get

$$E_\mathrm{g} = -N\Gamma + \frac{1}{2}\left(\sum_i A_{ii} - \sum_k \lambda_k\right) = -\frac{1}{2}\sum_k \lambda_k \ . \tag{15}$$

If we require that the $\mathbf{\Phi}_k$ are orthonormal, then the transformation given by Supplementary Eq. (7) is a canonical transformation. Solving Supplementary Eq. (10) gives the corresponding $\mathbf{\Psi}_k$.

Finding matrices Φ and Ψ (and hence g_{ik} and h_{ik}) gives the forward transform connecting the undiagonalized fermions to the diagonalized fermions. The inverse transform can be defined as

$$\begin{aligned} a_i^\dagger &= \sum_k \bar{g}_{ik}\eta_k^\dagger + \sum_k \bar{h}_{ik}\eta_k \\ a_i &= \sum_k \bar{g}_{ik}\eta_k + \sum_k \bar{h}_{ik}\eta_k^\dagger \ , \end{aligned} \tag{16}$$

such that

$$\begin{aligned} a_i^\dagger + a_i &= \sum_k \bar{\phi}_{ik}\left(\eta_k^\dagger + \eta_k\right) \\ a_i^\dagger - a_i &= \sum_k \bar{\psi}_{ik}\left(\eta_k^\dagger - \eta_k\right) \ , \end{aligned} \tag{17}$$

where $\bar{\phi}_{ik} = \bar{g}_{ik} + \bar{h}_{ik}$ and $\bar{\psi}_{ik} = \bar{g}_{ik} - \bar{h}_{ik}$. Since these transforms are canonical, $\bar{\Phi} = \Phi^{\mathrm{T}}$ and $\bar{\Psi} = \Psi^{\mathrm{T}}$.

Supplementary Note 2. FERMIONIC DOMAIN-WALL STATES

The eigenstates of the Hamiltonian can be rewritten as many-fermion states. For example, $|0\rangle$ denotes the vacuum which is the ground state of the Hamiltonian, $|a\ b\ c\rangle = \eta_a^\dagger\eta_b^\dagger\eta_c^\dagger\,|0\rangle$ is a three-fermion state with energy $E_{\mathrm{g}} + \lambda_a + \lambda_b + \lambda_c$ and $|\gamma\rangle = \eta_{\{\gamma\}}^\dagger\,|0\rangle$ is another state with $|\gamma|$ fermions. In this notation $|a\ b\ c\rangle$ means a state with a single fermion in each of the positions a, b, and c. What do these states look like in the computational basis? We can gain some intuition by considering the special case with zero transverse field.

For $\Gamma = 0$, Supplementary Eq. (12) becomes:

$$\mathbf{\Phi}_k \begin{pmatrix} 0 & & & & \\ & 4J_1^2 & & & \\ & & 4J_2^2 & & \\ & & & \ddots & \\ & & & & 4J_{N-1}^2 \end{pmatrix} = \lambda_k^2\mathbf{\Phi}_k \ . \tag{18}$$

For simplicity, assume $J_1 \le J_2 \le \ldots \le J_{N-1}$. This immediately yields the eigenvalues $\lambda_1 = 0$, $\lambda_2 = 2J_1$, $\lambda_3 = 2J_2$, …, $\lambda_N = 2J_{N-1}$ and eigenvector

$$\Phi = \begin{pmatrix} 1 & & & & \\ & 1 & & & \\ & & 1 & & \\ & & & \ddots & \\ & & & & 1 \end{pmatrix} \ . \tag{19}$$

Using Supplementary Eq. (11) we find:

$$\Psi = \begin{pmatrix} 0 & 0 & 0 & \ldots & 1 \\ -1 & 0 & 0 & \ldots & 0 \\ 0 & -1 & 0 & \ldots & 0 \\ 0 & & \ddots & & 0 \\ 0 & 0 & \ldots & -1 & 0 \end{pmatrix} \ . \tag{20}$$

If the J_i are not ordered the result remains the same up to a permutation of rows of Φ and Ψ, where the λ_i are still arranged in ascending order.

Now consider the operator $\sigma_i^z\sigma_{i+1}^z = (a_i^\dagger - a_i)(a_{i+1}^\dagger + a_{i+1})$ in terms of the diagonalized fermionic operators. Using Supplementary Eqs. (17), (19) and (20) gives

$$\begin{aligned} \sigma_i^z\sigma_{i+1}^z &= \sum_{kk'} \bar{\psi}_{ki}\bar{\phi}_{i+1,k'}(\eta_k^\dagger - \eta_k)(\eta_{k'}^\dagger + \eta_{k'}) \\ &= \sum_{kk'} (-\delta_{k,i+1})(\delta_{k',i+1})(\eta_k^\dagger - \eta_k)(\eta_{k'}^\dagger + \eta_{k'}) \\ &= 1 - 2\eta_{i+1}^\dagger\eta_{i+1} \ . \end{aligned} \tag{21}$$

Thus, the many-fermion states are also the eigenstates of the operators $\sigma_i^z\sigma_{i+1}^z$ with eigenvalue -1 if there is a fermion occupying level $i+1$, and eigenvalue 1 otherwise. In the spin picture, eigenvalue $+1$ denotes a satisfied coupling and -1 denotes an unsatisfied coupling. Thus, the presence of a fermion in level $i+1$ can be thought as a domain wall in coupling i of the ferromagnetic chain. The fermionic states $|0\rangle$ and $|1\rangle$ satisfy all the couplings and hence are linear combinations of the all-0 and all-1 states. This is reflective of the $\mathbb{Z}_2$ symmetry inherent in the Ising Hamiltonian, which also manifest itself as $\lambda_1 = 0$. Thus, for given state $|a\rangle$ with energy $E_a = E_{\mathrm{g}} + \lambda_a$, the state $|a\ 1\rangle$ has the same energy $E_{\mathrm{g}} + \lambda_a + \lambda_1 = E_a$. Additionally, $(1 - 2\eta_{i+1}^\dagger\eta_{i+1})\,|a\rangle = (1 - 2\eta_{i+1}^\dagger\eta_{i+1})\,|a\ 1\rangle \ \ \forall\ i \ge 1$ since the fermion occupying the state $|1\rangle$ is not affected by this operation. Ergo, the states $|2\rangle$ and $|2\ 1\rangle$ corresponds to a domain wall at the location of the weakest coupling, $|3\rangle$ and $|3\ 1\rangle$ corresponds to a state with a domain wall at the second weakest coupling, etc. Once the couplers are arranged in alternating sectors such that $J_1 = J_2 = \ldots = W_1 > J_{n+1} = J_{n+2} = \ldots = W_2 < \ldots$, the domain walls first occur in the light sectors, followed by the heavy sectors, etc.

Supplementary Note 3. WHY STATES THAT DIFFER BY A SINGLE FERMIONIC EXCITATION HAVE THE LARGEST MATRIX ELEMENTS

In order to determine whether thermal transitions between states can occur, we need to calculate the matrix element of σ^z between the two states, where we assume that the dominant interaction term between the system and environment is given by a pure dephasing interaction of the form $H_{\mathrm{SB}} = \sum_i \sigma_i^z \otimes B_i$ [2–5]. We we would like to estimate the transition matrix element $\langle\gamma_1|\sigma_i^z|\gamma_2\rangle$ between states $|\gamma_1\rangle$ and $|\gamma_2\rangle$. We shall show that these transitions are most prominent when the states differ by only a single fermion.

In terms of the fermionic operators, the operator σ^z can be written as

$$\sigma_i^z = \left[\prod_{j=1}^{i-1}(a_j^\dagger + a_j)(a_j^\dagger - a_j)\right](a_i^\dagger + a_i) \tag{22}$$

and it can in turn be written in term of the $\{\eta_k\}$ and $\{\eta_k^\dagger\}$ operators using Supplementary Eq. (17).

Now consider the matrix element between the vacuum state $|0\rangle$ and the state $|\gamma\rangle$. For the operator σ_1^z we find that

$$\begin{aligned}\langle\gamma|\sigma_1^z|0\rangle &= \langle\gamma|a_1^\dagger + a_1|0\rangle \\ &= \langle\gamma|\sum_k \bar{\phi}_{1k}(\eta_k^\dagger + \eta_k)|0\rangle \\ &= \sum_k \bar{\phi}_{1k}\,\langle\gamma|k\rangle\,.\end{aligned} \tag{23}$$

This can only be non-zero if $|\gamma\rangle$ is a single fermion state. Thus, the operator σ_1^z connects the vacuum to single-fermion states.

For σ_2^z, we have

$$\begin{aligned}&\langle\gamma|\sigma_2^z|0\rangle = \langle\gamma|(a_1^\dagger + a_1)(a_1^\dagger - a_1)(a_2^\dagger + a_2)|0\rangle \\ &= \sum_{k,l,m} \bar{\phi}_{1k}\bar{\psi}_{1l}\bar{\phi}_{2m}\,\langle\gamma|(\eta_k^\dagger + \eta_k)(\eta_l^\dagger + \eta_l)(\eta_m^\dagger + \eta_m)|0\rangle\,.\end{aligned} \tag{24}$$

For the above term to be non-zero, the state $|\gamma\rangle$ must be either a three-fermion or a single-fermion state. The one-fermion case can be computed in a similar manner to the previous case involving σ_1^z, so we focus on the case when $|\gamma\rangle = |a\ b\ c\rangle$ is a three-fermion state. We have

$$\begin{aligned}&\langle a\ b\ c|\sigma_2^z|0\rangle \\ &= \sum_{klm} \bar{\phi}_{1k}\bar{\psi}_{1l}\bar{\phi}_{2m}\,\langle a\ b\ c|\eta_k^\dagger\eta_l^\dagger\eta_m^\dagger|0\rangle \\ &= \sum_{klm} \bar{\phi}_{1k}\bar{\psi}_{1l}\bar{\phi}_{2m}\,\langle a\ b\ c|k\ l\ m\rangle \\ &= \det\begin{vmatrix}\bar{\phi}_{1a} & \bar{\psi}_{1a} & \bar{\phi}_{2a}\\ \bar{\phi}_{1b} & \bar{\psi}_{1b} & \bar{\phi}_{2b}\\ \bar{\phi}_{1c} & \bar{\psi}_{1c} & \bar{\phi}_{2c}\end{vmatrix}\,.\end{aligned} \tag{25}$$

We find numerically for our problems that the matrix element associated with the three-fermion states is much smaller than that for single-fermion states. For example, for a chain of length $N = 176$ with parameters $n = 5$, $W_1 = 1$ and $W_2 = 0.5$, we find at $s = s^*$ that $\langle 3|\sigma_1^z|0\rangle = 0.12$ and $\langle 2\ 3\ 4|\sigma_2^z|0\rangle = 1.2\times 10^{-8}$.

Similarly, the matrix element involving σ_3^z requires $|\gamma\rangle$ to be a state with 1, 3 or 5 fermions, etc. We find that the excitation to the 5-fermion states will be even smaller than that to the 3-fermion states, since they contain terms that are the product of five terms of the type $\bar{\phi}\bar{\psi}\bar{\phi}\bar{\psi}\bar{\phi}$. Thus, our numerical results indicate that the vacuum state couples predominantly to single-fermion states.

The above analysis can be generalized. Let us consider the matrix element $\theta_{ij} = \langle 0|\sigma_i^z|j\rangle$. Let $\Theta = [\theta_{ij}]$ and $\bar{\Psi} = [\bar{\psi}_{ij}]$, and consider $\tilde{G} = \Theta\,\bar{\Psi}^{\mathrm{T}}$. We have:

$$\tilde{G}_{ij} = \sum_m \theta_{im}\left(\bar{\Psi}^{\mathrm{T}}\right)_{mj} \tag{26}$$
$$= \sum_m \bar{\psi}_{jm}\,\langle 0|\sigma_i^z|m\rangle \tag{27}$$
$$= \langle 0|\sigma_i^z\left(\sum_m \bar{\psi}_{jm}(\eta_m^\dagger - \eta_m)\right)|0\rangle \tag{28}$$
$$= \langle 0|\sigma_i^z(a_j^\dagger - a_j)|0\rangle \tag{29}$$
$$= \langle 0|A_1B_1\ldots A_{i-1}B_{i-1}A_iB_j|0\rangle\ , \tag{30}$$

where we have defined $A_i = (a_i^\dagger + a_i)$ and $B_i = (a_i^\dagger - a_i)$. Since the operators A_i and B_j in Supplementary Eq. (30) anticommute when they have different indices, we can simplify the expression using Wick's theorem [1, 6]. For a set of anticommutating operators $\{O_1, O_2, \ldots, O_{2n}\}$, Wick's theorem states that

$$\begin{aligned}&\langle 0|O_1O_2\ldots O_{2n}|0\rangle \\ &\quad= \sum_{\text{possible pairings}} (-1)^p \prod_{\text{all pairs}} \langle 0|O_{i_1}O_{i_2}|0\rangle\ .\end{aligned} \tag{31}$$

where the sum is over all possible pairings of the operators $\{O_1, O_2, \ldots, O_{2n}\}$, the product is over the two-point expectation value of all pairs, and $(-1)^p$ is the sign of the permutation that is required to bring the paired terms next to each other. For an odd number of operators, the expectation value vanishes. Applying the theorem to Supplementary Eq. (30), we can make the following simplifications:

$$\begin{aligned}\langle 0|A_iA_j|0\rangle &= \sum_k \bar{\phi}_{ik}\bar{\phi}_{jk} = \delta_{ij}\ ,\\ \langle 0|B_iB_j|0\rangle &= -\sum_k \bar{\psi}_{ik}\bar{\psi}_{jk} = -\delta_{ij}\ ,\\ \langle 0|A_iB_j|0\rangle &= \sum_k \bar{\phi}_{ik}\bar{\psi}_{jk} = \left(\bar{\Phi}\,\bar{\Psi}^{\mathrm{T}}\right)_{ij} \equiv G_{ij}\ .\end{aligned} \tag{32}$$

If $i < j$, all the terms in Supplementary Eq. (30) will have different indices. The non-zero terms are pairs of the form $\langle A_kB_{k'}\rangle$. An obvious pairing is $\langle A_1B_1\rangle\ldots\langle A_iB_j\rangle = G_{11}G_{22}\ldots G_{ij}$ and all other permutations can be obtained by keeping the A's fixed and permuting the B's around them. The signature of the permutation will be the signature of the permutations of B's. The sum over permutations p is then given by

$$\begin{aligned}\tilde{G}_{ij} &= \sum_p (-1)^p G_{1p_1}G_{2p_2}\ldots G_{ip_i} \\ &= \det\begin{vmatrix}G_{11} & G_{12} & \ldots & G_{1j}\\ G_{21} & G_{22} & \ldots & G_{2j}\\ \vdots & & & \\ G_{i1} & G_{i2} & \ldots & G_{ij}\end{vmatrix}\,.\end{aligned} \tag{33}$$

If $i \geq j$, we need to further simplify Supplementary Eq. (30) so that it only contains anticommuting terms;

$$\begin{aligned}\langle A_1B_1\dots A_jB_j\dots A_iB_j\rangle &= -\langle A_1B_1\dots A_jB_jB_j\dots A_i\rangle\\ &= -\langle A_1B_1\dots A_j(-1)\dots A_i\rangle\\ &= \langle A_1B_1\dots A_j\dots A_{i-1}B_{i-1}A_i\rangle\ .\end{aligned} \quad (34)$$

In Wick's expansion of this equation, any possible permutation will contain a pair of form $\langle A_k A_{k'}\rangle = 0$. Thus, $\tilde{G}_{ij} = 0$ for $i > j$.

This gives us a method to calculate the matrix elements of $\tilde{G}$, and we can in turn compute the desired transition element matrix via

$$\Theta = \tilde{G}\bar{\Psi}\ . \quad (35)$$

Matrix element between arbitrary states can be computed in a similar fashion. For example:

$$\begin{aligned}\langle\gamma_1|\sigma_i^z|\gamma_2\rangle &= \langle\gamma_1|A_1B_1\dots A_{i-1}B_{i-1}A_i|\gamma_2\rangle\\ &= \langle\gamma_1|A_1B_1\dots A_{i-1}B_{i-1}A_i\eta^\dagger_{\{\gamma_2\}}|0\rangle\ .\end{aligned} \quad (36)$$

Since $A_i = \sum_j \bar{\phi}_{ij}(\eta_j^\dagger + \eta_j)$ and $B_i = \sum_j \bar{\psi}_{ij}(\eta_j^\dagger - \eta_j)$ we have

$$\begin{aligned}\{A_i, \eta_j^\dagger\} &= \bar{\phi}_{ij}\ ,\\ \{B_i, \eta_j^\dagger\} &= -\bar{\psi}_{ij}\ .\end{aligned} \quad (37)$$

We can therefore anticommute the creation operator of $\eta^\dagger_{\{\gamma_2\}}$ from the left hand side to the right hand side. We find that the additional terms appearing because of the anticommutation relation in Supplementary Eq. (37) are small relative to the term proportional to $\langle\gamma_1|\eta^\dagger_{\{\gamma_2\}}A_1B_1\dots A_{i-1}B_{i-1}A_i|0\rangle$, so we focus only on this term:

$$\begin{aligned}&\langle\gamma_1|\sigma_i^z|\gamma_2\rangle\\ &\sim (-1)^{(2i-1)|\gamma_2|}\langle\gamma_1|\eta^\dagger_{\{\gamma_2\}}A_1B_1\dots A_{i-1}B_{i-1}A_i|0\rangle\\ &= (-1)^{(2i-1)|\gamma_2|}\langle\gamma_1-\gamma_2|A_1B_1\dots A_{i-1}B_{i-1}A_i|0\rangle\\ &= (-1)^{(2i-1)|\gamma_2|}\langle\gamma_1-\gamma_2|\sigma_i^z|0\rangle\ ,\end{aligned} \quad (38)$$

where $(-1)^{(2i-1)|\gamma_2|}$ is an overall phase term associated with anticommuting the set $\eta^\dagger_{\{\gamma_2\}}$ to the left and $|\gamma_1-\gamma_2\rangle = \eta_{\{\gamma_2\}}|\gamma_1\rangle$. For this term to be non zero, the state $|\gamma_1\rangle$ should contain all the fermions found in the state $|\gamma_2\rangle$. The remaining quantity $\langle\gamma_1-\gamma_2|\sigma_i^z|0\rangle$ is a matrix element connecting the vacuum, which we have already argued is largest when the state $|\gamma_1-\gamma_2\rangle$ is a single-fermion state. Therefore, the matrix elements connecting $|\gamma_1\rangle$ and $|\gamma_2\rangle$ are largest when the two states differ only by one fermion.

From the above analysis of the coupling matrix elements, we find that the ground state couples principally with the single-fermion states, and the single-fermion states couple principally to two-fermion states and so on. The transition energies are the single-fermion energies $\{\lambda_i\}$ found by solving the eigensystem of Supplementary Eq. (12).

Supplementary Note 4. EXPONENTIAL DEPENDENCE OF THE SUCCESS PROBABILITY ON k^*

In this section we provide a more detailed argument than given in the main text for why the success probability exhibits an exponential dependence on k^*. Rather than providing a counting argument based on the thermal density of states d, as in Article's Eq. (7), we consider the transition rates appearing in the Pauli master equation. Our argument provides a justification for why, if it were numerically feasible, we would expect the master equation simulations to reproduce the exponential dependence on k^* seen in our empirical results.

Given the form of the Pauli master equation [Article's Eq. (8)], $\dot{p}_a = \sum_{b\neq a}\gamma(\omega_{ba})M_{ab}p_b - \left(\sum_{b\neq a}\gamma(\omega_{ab})M_{ba}\right)p_a$, where [Article's Eq. (9)] $M_{ab} = \sum_\alpha |\langle a|A_\alpha|b\rangle|^2$, we expect the success probability to be inversely related to the overall excitation rate. Let $M_{0b} \equiv M_b$ be the matrix element involving a transition between states via the creation or annihilation of a fermion with energy λ_b, where $\lambda_b \leq \lambda_{k^*}$. Let the number of such connected states be $\#_b = \binom{k^*}{b}$ and let $M_{\min}$ ($M_{\max}$) be the minimum (maximum) matrix element within the set $\{M_1, M_2, \dots, M_{k^*}\}$. The total transition rate τ is can be estimated as

$$\tau \sim M_1\times\#_1 + M_2\times\#_2 + \dots + M_{k^*}\times\#_{k^*}\ , \quad (39)$$

so that

$$M_{\min}2^{k^*} \lesssim \tau \lesssim M_{\max}2^{k^*}\ , \quad (40)$$

where we used $\sum_{b=0}^{k^*}\#_b = 2^{k^*}$. This bound is meaningful if the matrix elements do not differ by orders of magnitude. Indeed, we found numerically that the matrix elements are within an order of magnitude of each other. For example, for the chain with parameters $N = 176$, $n = 5$, $W_1 = 1$ and $W_2 = 0.5$, we found that at the critical point with $k^* = 18$, the largest matrix element is $M_{\max} = M_1 = 43.76$ and the smallest matrix element is $M_{\min} = M_{18} = 0.99$. The difference in these values is small compared to the number of fermionic states, which is 2^{18}. Thus, $\tau = \Omega(2^{k^*})$ and we expect the success probability to be inversely proportional to 2^{k^*}.

Supplementary Note 5. TEST OF THE ADIABATIC CONDITION

Here we test the validity conditions assumed for the derivation of the adiabatic Markovian master equation [7], and in particular that the adiabatic approximation is satisfied. To this end we test the 'folklore' adiabatic condition $t_f \gg \max_s |\langle g|\dot{H}(s)|e\rangle|/\Delta^2(s)$, where g and e are the ground and first (relevant) excited states, respectively, for ASC parameters $(1, 0.5, 175)$. The result is shown in Supplementary Fig. 1 (red circles), and it can

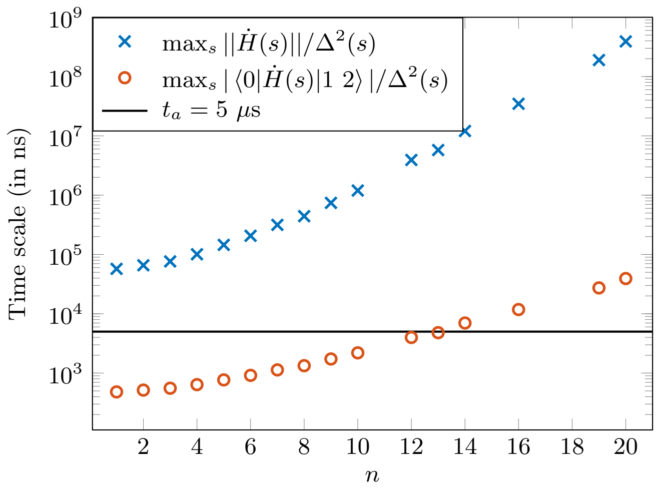

Supplementary Figure 1. **Test of the adiabatic condition**. The solid line is the annealing time used in our experiments. Symbols represent the quantity appearing in two versions of the adiabatic condition [for ASC parameters $(1, 0.5, 175)$] that should be smaller than the annealing time in order for the adiabatic condition to hold.

Chain parameters	DW2X	k^*	ME	SVMC
$(1.0, 0.50, 175)$	5,6	5	5,6	5
$(0.5, 0.25, 175)$	3,4,5	4	4,5	5,6,7
$(0.8, 0.40, 175)$	5	5	5	5,6
$(1.0, 0.50, 200)$	5,6	5	5,6	4,5,6
$(1.0, 0.50, \ \ 55)$	5,6	5	5,6,8	5
$(0.6, 0.30, 135)$	4,5	4,5	5,6	5,6

Supplementary Table I. Locations n^* of the DW2X success probability minima vs those found by the fermionic model based on the peak of k^*, the master equation model (ME), and the SVMC model. When the location of the minimum is ambiguous within our 95% confidence interval we list all values of n^* that overlap to within one σ. The best agreement is obtained by the master equation model.

be seen that the condition is satisfied for $n \leq 14$. The relevant first excited state is the two-fermion state $|1\ 2\rangle$. Also shown is the more conservative condition given in terms of the operator norm (blue crosses). The adiabatic condition with the operator norm is not satisfied for any value of n, but it has recently been shown that this condition, which involves an extensively growing operator norm, must be replaced by a condition involving local operators [8].

Supplementary Note 6. COMPARISON TO THE CLASSICAL SVMC MODEL

The spin vector Monte Carlo (SVMC) model [9] was proposed as a purely classical model of the D-Wave processors in response to earlier work that ruled out simulated annealing [10] and other work that established a strong correlation between D-Wave ground state success probability data and simulated quantum annealing [11]. The SVMC model was found to not always correlate well with D-Wave empirical data; for example, deviations were observed for the SVMC model in the case of ground state degeneracy breaking [4], excited state distributions [5], quantum annealing correction experiments [12] and the dependence of success probability on temperature [3]. But it has generally been successful in predicting the success probability distributions of D-Wave experiments. The SVMC model thus provides a sensitive test for whether anything other than classical effects are at play in a fixed-temperature measurement of the ground state success probability.

In the SVMC model each qubit is replaced by a classical $O(2)$ rotor parametrized by one continuous angle via $\sigma_i^x \mapsto \sin\theta_i$ and $\sigma_i^z \mapsto \cos\theta_i$, so that the Article's Hamiltonian (1) becomes

$$E(s) = -A(s)\sum_i \sin\theta_i + B(s)\left(-\sum_i J_i \cos\theta_i \cos\theta_{i+1}\right) . \tag{41}$$

In addition, the angles θ_i undergo Metropolis updates every discrete time-step of size $1/N_s$, where N_s is the number of sweeps, at a fixed inverse temperature β.

Angle updates are performed as follows. We first divide the dimensionless time s into steps of size $\delta s = 1/N_s$, where N_s is the number of sweeps performed during the course of one run, and initialize to the state $\theta_i(s=0) = \pi/2$ for all spins i. At each such time step, we pick a random permutation of the set $\{1, 2, \ldots, N\}$ where N is the number of qubits in the chain. One by one, we select a random angle for each qubit in this permuted list, changing it from θ_i to $\tilde{\theta}_i$ where $\tilde{\theta}_i$ is picked randomly from $[0, \pi]$. We calculate the energy change ΔE for this move and the new angle is accepted with probability

$$p_i = \min\left[1, \exp(-\beta\Delta E)\right] , \tag{42}$$

where β is the inverse annealing temperature for the SVMC algorithm. The final state is obtained by projecting the $O(2)$ rotor spins to the computational state at $s = 1$, setting spin i to be 1 (-1) if $\cos\theta_i > 0$ (< 0). We repeat this process many times in order to estimate the success probability of the algorithm.

In order to realistically emulate the D-Wave processor, we added random Gaussian noise $\mathcal{N}(0, \sigma^2)$ to each J_i [13]. The SVMC model then has three free parameters: $\{N_s, \beta, \sigma\}$, which we used to calibrate it against the DW2X data. Toward this end we used the chain with parameters $(1, 0.5, 175)$ and performed an extensive search in the $\{N_s, \beta, \sigma\}$ parameter space. As shown in Supplementary Fig. 2(a), we obtain a close match for $N_s = 120 \times 10^3$, $\beta = 0.75$ (GHz)$^{-1}$ and $\sigma = 0.05$. This is the best fit we found for this particular chain. In general, we found that the SVMC parameters can be tuned to reproduce the location of the minimum in success probability for any sector size. We were also able to tune

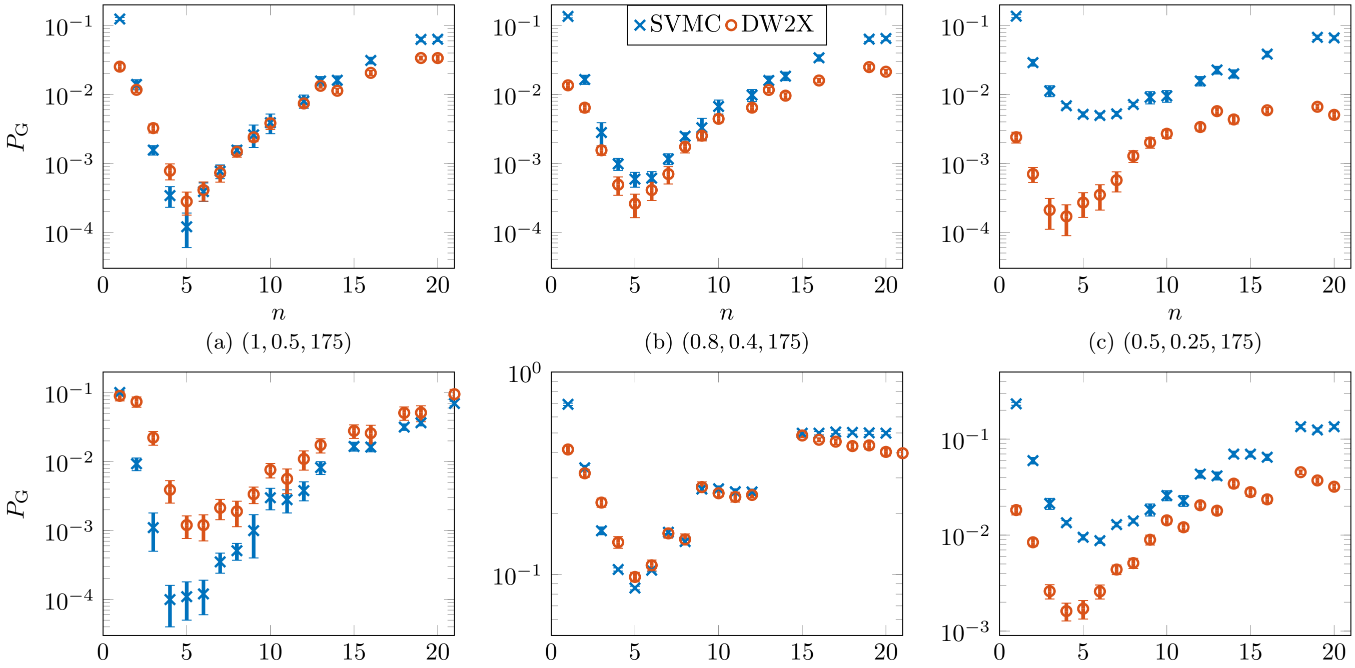

(a) (1, 0.5, 175)

(b) (0.8, 0.4, 175)

(c) (0.5, 0.25, 175)

(d) (1, 0.5, 200)

(e) (1, 0.5, 55)

(f) (0.6, 0.3, 135)

Supplementary Figure 2. **Comparison of the SMVC model to the empirical DW2X results**. The error bars everywhere indicate 95% confidence intervals calculated using a binomial bootstrap over the different runs of the simulation. (a) The SVMC parameters were optimized to match the empirical DW2X success probability results for the chain with parameters $(W_1, W_2, N) = (1, 0.5, 175)$. The optimal values found are: $N_s = 120 \times 10^3, \beta = 0.75$ (GHz)$^{-1}, \sigma = 0.05$ [compare to the DW2X's $t_f = 5$ μs, $\beta = 0.637$ (GHz)$^{-1}, \sigma \sim 0.03$]. (b) With the same optimal SVMC parameter values, but with chain parameters $(0.8, 0.4, 175)$, the SVMC model predicts increased success probability, in contrast to the empirical results. The same trend continues but is more pronounced in (c), with additionally the position of the minimum shifting to the wrong location ($n = 7$ vs $n^* = 4$). Panel (d) shows that increasing the chain length causes a large deviation in the SVMC results [compare to panel (a)], and also shifts the location of the minimum to the wrong value, but (e) shows showing that reducing the chain length does not degrade the agreement much. (f) Results for another chain parameter set, exhibiting a similar discrepancy to that seen in (c).

the parameters such that the minimum disappears completely and have the success probability increase or decrease monotonically. We were not able to find parameters that give rise to an inverted curve, i.e., a maximum in the success probability. Most of these features can be seen by tuning β and keeping the other parameters fixed.

To avoid fine-tuning, we next used the same parameters to compute the success probability of the SVMC model for other chains. As shown in Supplementary Figs. 2(b) and 2(c), the SVMC model has the wrong trend with decreasing (W_1, W_2): it exhibits a higher success probability as the coupling energy scale is lowered. The same happens with increased chain length [Supplementary Fig. 2(d)], though to a lesser degree with decreased chain length [Supplementary Fig. 2(e)]. Moreover, as summarized in Table I, it does not agree as well with the location of the minimum of the success probability as the other models. We emphasize that while we performed an extensive search, we cannot rule out the possibility of another set of parameters (or the inclusion of other parameters) that allow SVMC to reproduce the DW2X results for all chain lengths and energy scales.

As a further test we also considered the spin boundary correlation, defined as the sum of spin correlations over all boundary qubits in the chain, where the boundary qubits are the qubits at the right edge (r) of the heavy sector and left edge (l) of the light sector:

$$\bar{s} = \frac{1}{|Q|} \sum_{Q} s_l s_r \ , \tag{43}$$

where Q is the set of boundary qubits and $s_l, s_r \in \{0, 1\}$. Thus $\bar{s} = 1$ represents perfect alignment (a ground state), while $\bar{s} < 1$ represents the occurrence of an excited state due to misalignment of the different sectors. Supplementary Fig. 3 shows the results for the same set of optimized parameters that provided strong agreement with the ground state success probability in Supplementary Fig. 2(a). It can be seen that the SVMC model predicts the wrong location for the minimum of $\bar{s}$ and rises too fast. Unfortunately, master equation simulations for $\bar{s}$ are numerically prohibitive, so we cannot assess whether

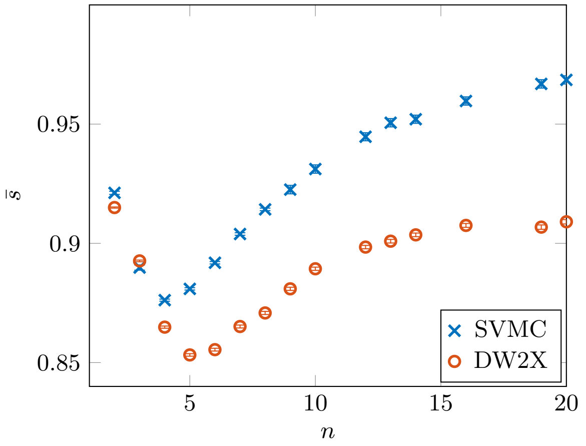

Supplementary Figure 3. **Spin boundary correlation function computed using the SVMC model.** The spin boundary correlation function for the same chain and SVMC parameters as in Supplementary Fig. 2(a). The SVMC model does not correctly capture the empirical results despite providing a close match to the success probability in Supplementary Fig. 2(a).

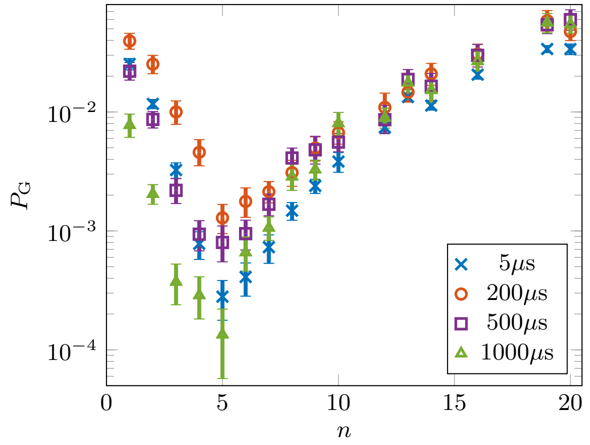

Supplementary Figure 4. **Dependence of the success probability on the annealing times.** We show the results for the ASC with parameters $(1.0, 0.5, 175)$. The error bars everywhere indicate 95% confidence intervals calculated using a bootstrap over different gauges and embeddings. The location of the minimum is unchanged as the annealing time is varied.

this discrepancy of the SVMC model is fixed by a quantum model.

Supplementary Note 7. RESULTS AT DIFFERENT ANNEALING TIMES

In Supplementary Note 5 we discussed the validity of the "folklore" adiabatic condition for the ASC problem. We expect that the adiabatic condition will also be satisfied if we make small changes in the annealing time compared to the vertical scale of Supplementary Fig. 1. Also, neither the gap Δ nor the number of single fermion states k^* changes with an increase in the annealing time. Hence we expect that the qualitative nature of the success probability curve, including the location of minima, will be independent of small changes in the annealing time. Since the total thermal transition rate depends on the amount of time system spends near the quantum minimum gap point s^*, we do expect to see changes in the value of the success probability. In Supplementary Fig. 4, we show the change in success probability as we vary the annealing time on the D-Wave device. As expected, the location of the minima remains unchanged. We do find that the success probability varies depending on the annealing time and sector size.

SUPPLEMENTARY REFERENCES

[1] Lieb, E., Schultz, T. & Mattis, D. Two soluble models of an antiferromagnetic chain. *Annals of Physics* **16**, 407–466 (1961).
[2] Lanting, T. *et al.* Cotunneling in pairs of coupled flux qubits. *Physical Review B* **82**, 060512 (2010).
[3] Boixo, S. *et al.* Computational multiqubit tunnelling in programmable quantum annealers. *Nature Communications* **7**, 10327 (2016).
[4] Albash, T., Vinci, W., Mishra, A., Warburton, P. A. & Lidar, D. A. Consistency tests of classical and quantum models for a quantum annealer. *Physical Review A* **91**, 042314 (2015).
[5] Albash, T., Hen, I., Spedalieri, F. M. & Lidar, D. A. Reexamination of the evidence for entanglement in a quantum annealer. *Phys. Rev. A* **92**, 062328 (2015).
[6] Wick, G. C. The Evaluation of the Collision Matrix. *Physical Review* **80**, 268–272 (1950).
[7] Albash, T., Boixo, S., Lidar, D. A. & Zanardi, P. Quantum adiabatic Markovian master equations. *New Journal of Physics* **14**, 123016 (2012).
[8] Bachmann, S., De Roeck, W. & Fraas, M. Adiabatic Theorem for Quantum Spin Systems. *Phys. Rev. Lett.* **119**, 060201 (2017).
[9] Shin, S. W., Smith, G., Smolin, J. A. & Vazirani, U. How "quantum" is the D-Wave machine? Preprint at http://arxiv.org/abs/1401.7087 (2014).
[10] Boixo, S., Albash, T., Spedalieri, F. M., Chancellor, N. & Lidar, D. A. Experimental signature of programmable quantum annealing. *Nat. Commun.* **4**, 2067 (2013).
[11] Boixo, S. *et al.* Evidence for quantum annealing with more than one hundred qubits. *Nat. Phys.* **10**, 218–224 (2014).
[12] Pudenz, K. L., Albash, T. & Lidar, D. A. Quantum annealing correction for random Ising problems. *Phys. Rev. A* **91**, 042302 (2015).
[13] Shin, S. W., Smith, G., Smolin, J. A. & Vazirani, U. Comment on "Distinguishing classical and quantum models for the D-Wave device". Preprint at http://arxiv.org/abs/1404.6499 (2014).
